# RETRACTION: Macrophage Deficiency Makes Intestinal Epithelial Cells Susceptible to NSAID‐Induced Damage

**DOI:** 10.1155/bmri/9789812

**Published:** 2026-04-03

**Authors:** BioMed Research International

RETRACTION: X. Wang, J. Wang, T. Xie, S. Li, D. Wu, Y. Lu, X. Guo, L. Chen, “Macrophage Deficiency Makes Intestinal Epithelial Cells Susceptible to NSAID‐Induced Damage,” *BioMed Research International*, 2020, 10.1155/2020/6757495

The above article, published online on 17 November 2020 in Wiley Online Library (http://wileyonlinelibrary.com), has been retracted by agreement between the journal Section Editor, Jinsong Ren, and John Wiley & Sons Ltd. The retraction has been agreed due to issues identified in the Figures, also raised on PubPeer [1]. Specifically:

• The TUNEL/Csf1op/op+NSAID panel of Figure 1a appears identical to the Ki67/Csf1op/op panel in Figure 10a>
• The claudins blots shown in Figure 2 appear to be the same as the right hand side HNF1A blots shown in Figure 2b of [2]
• Parts of the GAPDH blots shown in Figure 2 appear to be the same as parts of the GAPDH blots shown in Figure 1c of [2]
• The second (LPS + Caco‐2 + RAW264.7) flow cytometry plot shows similarities to:
  o. The bottom left (0G/CD133+) flow cytometry plot in Figure 2d of [3]
  o. The middle (PLT) flow cytometry plot in Figure 2a of [4]
• The staining images in Figure 8 show overlap in the majority of panels when compared to the following articles:
  o. Seven panels overlap with [5]
  o. The second migration panel in Figure 8 overlaps with the Migration/PLT panel in Figure 7b of [4]
  o. 3 panels overlap with [6]
  o. 4 panels overlap with [7]
  o. The GAPDH blots in Figure 9 appear similar to the right hand t‐AKT blots shown in Figure 4b of [8]
• The WT/TUNEL panel in Figure 1a appears the same as the first KYSE30/Vector panel in Figure 10c of [9]
• The Ki‐67/Csf1^op/op^ panel in Figure 1a appears the same as the first KYSE150/Vector panel in Figure 10c of [9]

The authors did not provide a response to the concerns or to the retraction.
